# On the Treatment of Prolapsus Uteri by the Local Application of Tannin

**Published:** 1857-11

**Authors:** G. A. Kunkler

**Affiliations:** Madison, Ind.


### ART. IV.—*On the Treatment of Prolapsus Uteri by the Local Application of Tannin.* By G. A. KUNKLER, M.D., of Madison, Ind.

EVERY scientific physician will admit that any practical and efficient suggestion in the treatment of such a distressing malady as the above, is a matter of interest to all branches of the profession; for cases such as are referred to are brought under the cognizance of us all, and I am sure that there are few who will not regard as a boon a method well adapted to fulfil that main object which we all have in view in treating a patient,—"a perfect cure." It is to the treatment by the local application of tannin in cases of simple descent of the womb, uncomplicated by disease, that I wish to direct attention.

The practice of treating prolapsus uteri by the local application of tannin, was, if I am not mistaken, first recommended by Dr. B. F. Barker, of New York, and has since been employed by several Eastern physicians with a very favorable result.

In a number of the New York Medical Times of the past year, Dr. Chas. A. Budd has published a paper, with the report of several cases, corroborative of the value of this treatment. The result in my own practice has proved so satisfactory, that in no future case shall I omit to give it a trial. I am convinced that in all cases where there exists an indication, the result will be satisfactory, provided it be persevered in by the patient as well as the physician. Among the cases in which this treatment is not applicable may be mentioned a loss of substance in the perineum, in consequence of laceration or other injuries, a very straight sacrum, &c. &c. The treatment is not so annoying to the patient as may be supposed, as any female attendant, or the patient herself, can change and reintroduce the lint whenever it is necessary.

The following cases may not prove without interest:

Case I. Mrs. C., aged 35, married, and without children, applied to me in June, 1856. She had been suffering with prolapsus uteri for twelve years. The patient is a stout, heavily-built woman, of a lymphatic temperament, and had previously always enjoyed good health. She attributes the disease to an abortion which she had in the sixth month of pregnancy; the placenta did not follow, and was forcibly removed. She has worn quite a number of different kinds of pessaries, but never was able to continue their use for any length of time, on account of the violent irritation, which they invariably caused. Menstruation has been very irregular for the past two years, generally returning every fifteen days, and occasionally to an alarming extent. At the time the patient came under my care, the whole body was bloated with anasarca; the uterus was somewhat enlarged and congested, and low down in the vagina; the cervix slightly protruding at the vulva. The organ could be reduced without much difficulty, but would descend immediately upon the erect posture being assumed.

The general health was at once attended to; bark, steel, wine, &c., and a light nutritious diet were ordered, and the recumbent posture enjoined. After the uterus was replaced, the vagina was ordered to be syringed three times daily with a solution of three drachms of gallic acid to a pint of water. About one week afterwards the tannin was applied. The manner in which I used the latter is the same as that recommended by Prof. Barker and Dr. Budd. A double or treble thickness of the common patent lint is taken, and a triangular portion is cut out of a sufficient size, when firmly rolled up so as to form a cone, to fill the capacity of the vagina; near the apex a string may be attached to facilitate its withdrawal. The patient is placed upon her back, with the hips slightly elevated, and after the uterus has been replaced the lint, soaked in a solution of one ounce of tannin to eight ounces of water, is introduced, after being firmly rolled up, with its apex downwards, and its base in immediate contact with the os tincæ. The solution should be used cold, and applied daily, for a longer or shorter period, in accordance with the extent of the displacement. In the above-mentioned case it was applied almost successively for about forty-two days; no unpleasant symptom was noticed as resulting from it. The patient is now in perfect health, and has, up to the present date, not the slightest symptom of a return of the disease, being able to attend to the most laborious domestic duties. The menses became regular at once, upon the removal of the disease. Constipation was rigidly guarded against by the exhibition of either castor oil or the colocynth and hyoscyamus pill.

Case II. Mrs. H., aged 22 years, the wife of a respectable clergyman, and mother of two children, has been affected with prolapsus uteri since her confinement with her first child about four years ago, and after confinement with her second child, about a year and a half ago, the symptoms returned in a still more aggravated degree. The patient is a feeble, delicate woman, and subject to the most severe and convulsive form of hysteria, somewhat resembling epilepsy. Digestion was depraved, the menses irregular and always attended with much suffering; the bowels were always constipated. The uterus was found very low in the vagina. The general health was corrected, and the gallic acid injections used preparatory to the application of the tannin; the latter was applied as in the preceding case, the recumbent posture being enjoined for nearly one month. At the expiration of this time, the uterus occupied its proper position; the leucorrhœa, with which she had been affected for a long time, had disappeared, and from that time the violent hysteric paroxysms never returned, the patient ever since enjoying perfect health.

Case III. Mrs. W., aged 34, the mother of three children, the youngest being seven years of age. The patient has been subject to prolapsus uteri since the birth of her last child, and attributes it to a rudely managed accouchement. She is of a nervous hysterical temperament, and a feeble, delicate constitution. There was continually a profuse leucorrhœal discharge, the menses were scanty and irregular, and the uterus very low in the vagina. Injections of a weak solution of alum were used for a short time, after which, the lint, soaked in the tannin solution, was applied daily, for about three weeks, when she was dismissed, relieved. This patient had worn several pessaries for a short time, but was always compelled to lay them aside, on account of the irritation which they produced, although they fitted well, and were properly constructed.

